# “Animal-Type Melanoma/Pigmented Epithelioid Melanocytoma”: History and Features of a Controversial Entity

**DOI:** 10.3390/dermatopathology8030033

**Published:** 2021-07-05

**Authors:** Gerardo Cazzato, Francesca Arezzo, Anna Colagrande, Antonietta Cimmino, Teresa Lettini, Sara Sablone, Leonardo Resta, Giuseppe Ingravallo

**Affiliations:** 1 Section of Pathology, Department of Emergency and Organ Transplantation (DETO), University of Bari “Aldo Moro”, 70124 Bari, Italy; anna.colagrande@gmail.com (A.C.); micasucci@inwind.it (A.C.); lettinit@yahoo.com (T.L.); Leonardo.resta@uniba.it (L.R.); 2 Section of Obstetrics and Gynecology, University of Bari Aldo Moro, 70124 Bari, Italy; francescaarezzo@libero.it; 3 Section of Legal Medicine, Department of Interdisciplinary Medicine, Bari Policlinico Hospital, University of Bari Aldo Moro, 70124 Bari, Italy; sarasabloneml@gmail.com

**Keywords:** animal-type, melanoma, skin, malignancies

## Abstract

Animal-type melanoma (ATM) was first described in the literature by Levene in 1979 in relation to a patient with a characteristic clinical presentation, and only later, rare and anecdotal case series have tried to shed light on an entity that has undergone several nosographic classification changes, and which, since 2018, is classified under the term “pigmented epithelioid melanocytoma”. Here, we conduct a brief review of the current literature on ATM and present a new clinical case with histopathological, immunophenotypic, and molecular investigations.

## 1. Introduction

Animal-type melanoma is a rare variant of malignant melanoma described for the first time in humans in 1979 by Levene [1], which took this name by virtue of alleged similarity with equine melanotic disease (in gray horses) [1,2]. The nomenclatures used over the years have been different and multiform, and the rarity of these entities has further complicated a situation that is already complex in itself [2,3]. Histologically, ATM is characterized by heavily melanized spindle, dendritic, and/or epithelioid cells and a deep solid and periappendageal growth pattern [4,5]. In the latest edition of the WHO Skin Tumors, ATM was classified as “pigmented epithelioid melanocytoma” (PEM) and it was strongly advised against using the term “animal-type melanoma” again, as it is a potential source of confusion [6]. Interestingly, in 2010, Ludgate et al. proposed a scheme that divides these lesions into “equivocal” and “unequivocal”, stressing the concept of how the rarity of this entity and of the non-stringent morphological criteria can determine difficulties in the definition, in the correct nosographic classification, and thus in the best therapeutic approach for the patient [7]. In this paper, it is our intention to discuss the history of this entity, to outline the classification changes that have occurred up to the most recent times, and to present a new case of ATM that we have been able to study over the last few months. Finally, in the discussion session, we briefly discuss the different positions taken by the various authors in the literature.

## 2. Materials and Methods

The review of the literature was conducted using Pubmed and Web of Science (WoS) search engines, referring to the following words: “animal-type melanoma” OR “pigmented epithelioid melanocytoma” OR “melanoma with prominent melanin synthesis” AND “skin “OR” cutaneous malignancies “. As for the presentation of the clinical case, the patient’s clinical history was retrieved, with the diagnosis accompanied by immunophenotypic and molecular information. Informed consent of the person or subjects involved in the study was requested and obtained. A 73-year-old man came to the dermatologist for observation after noticing for about 7 months the appearance of a strongly pigmented, blackish-brown nodule, positioned on the skin of the scalp (right parieto-occipital region), which had given rise to concern to the patient following traumatic rubbing bleeding. After the advancement of the clinical suspicion of “atypical melanocytic lesion”, the patient was sent to the plastic surgeon for surgical excision.

## 3. Results

**Figure 1:**
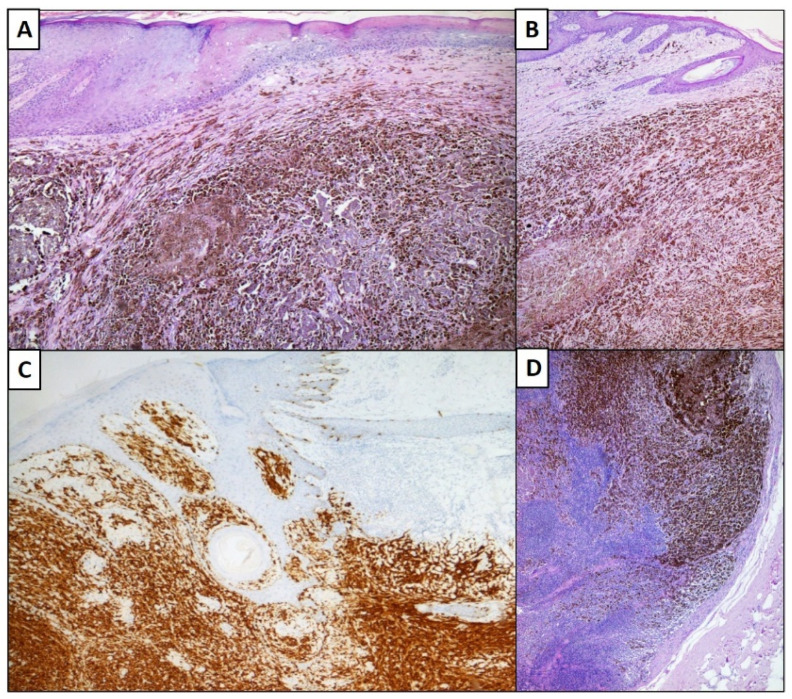
(**A**) Skin and subcutis including intensely pigmented melanocyte proliferation. (Hematoxylin-Eosin, Original Magnification: 4×). (**B**) Histological detail of melanocyte proliferation consisting of variously intertwined cells, with evident presence of melanic pigment. (Hematoxylin-Eosin, Original Magnification: 10×). (**C**) Immunohistochemical preparation with anti-Melan-A antibody which highlights the melanocyte proliferation in question (Immunohistochemistry, Original Magnification: 4×). (**D**) Detail of micrometastases in the sentinel lymph node (Hematoxylin-Eosin, Original Magnification: 4×).

Histopathological examination revealed an animal-type hyperpigmented spindle cell melanoma with extensive necrosis phenomena and the presence of numerous melanophages (Figure 1). The neoplasm entirely occupies the dermis and sometimes seemed to approach the subcutaneous tissue (V Clark Level). The growth pattern was predominantly in large nests, columns, and sometimes sheets of cells that tended to displace and destroy normal dermal and follicular skin structures (Figure 1A,B). The cells were predominantly spindle, sometimes epithelioid, and even more rarely dendritic (Figure 1B). The nuclei were vesicular with thickened nuclear membrane, and hyperchromatic and with thickened chromatin. It was also possible to appreciate large, irregular, and angulated nucleoli. Mitotic figures and necrosis were present. The thickness of Breslow was 25 mm, with an intratumoral and peritumor lymphocyte infiltrate completely absent, without evident signs of vascular invasion and neurotropism. Regression was also absent, but satellite phenomena were described (<2.0 cm from the primary lesion).

Immunostaining for Melan-A (Figure 1C), HMB-45, S-100 was strongly positive, fully confirming the morphological diagnosis.

A few weeks later, sentinel lymph node biopsy (BLNS) was performed, showing subcapsular and intraparenchymal metastases of melanoma referable to the primitive animal-type phenotype. The process was continued with right latero-cervical lymphadenectomy (Levels I-V), which was found to be negative for localization of the disease.

A study was subsequently carried out at another immunohistochemistry center for the protein kinase A regulatory subunit 1 alpha (PRKAR1A), which demonstrated negativity in melanoma cells. No GNAQ mutations were found.

## 4. Discussion

Over the years, there have been various nomenclatures to designate the entity animal-type melanoma (or melanoma with prominent melanin synthesis). In 1979, Levene [1] was the first author to describe such a case by reporting his experience with a patient who developed many pigmented blotches on the sclera and face and that, then, when he is an adult, he developed hepatomegaly, which proved to be due to metastatic melanoma. Indeed, autopsy showed blackening of the dura with infiltrates in the cerebral cortex and basal ganglia. Histological examination shows widespread deposition of melanophages in the body and in some organs. However, the primary tumor was never found in Levene’s patient. From Levene’s work onwards, the term animal-type melanoma began to spread in dermatopathology to designate this type of lesion, although it already seemed at the time that this nomenclature was too weak and too generic. More in detail, as several papers began to describe this entity in humans [2,3,4,5,6,7,8,9], it became clear that, in humans, it was less malignant than the homologous disease of gray horses [10], making this nomenclature more and more insufficient to define its salient features.

Mones and Ackerman, in 2004, stressed the concept of the correct differential diagnosis that must be placed between ATM and other lesions such as atypical blue nevus, melanocytoma, or melanoma with features of blue nevus and regressed melanoma with nodular melanosis [10].

In 2007, Raquena L. tried to deal with this topic, admitting that, even at the time, this entity was difficult to interpret and that there remained the problem of unclear and defined diagnostic criteria with respect to entities such as atypical blue nevus or melanoma arising on blue nevus [11]. Antony et al. [5] and Ludgate et al. [7] reported their serious cases (of 14 and 22 patients, respectively) through which they tried to delineate the clinical-biological behavior of the ATM: the first stated that this entity could be counted among the low-grade malignant neoplasms whose surgical excision with large margins could be considered decisive, while Ludgate et al., presenting their 22 cases, agreed on the fact that the behavior of this entity was varied—that, on average, the younger the subjects were, the better the clinical outcome, but that the presence of cases with aggressive behavior. We feel we can place our new case presented here in this category. Another point that seems very important to us to underline concerns the description by the latter authors [7] of a subset of ATM with mild cellular atypia and monomorphic morphology; this subgroup has been identified as “equivocal” ATM compared with the group with stringent cellular atypia identified as “unequivocal”. As already mentioned, in the latest WHO Skin Tumours, this entity has been merged under the definition of PEM, considering it in all respects as a variant of the blue epithelioid nevus. The term PEM was preferred to better reflect the intermediate malignant potential of this tumor [6,12]. Although these lesions have been mostly described in children, adolescents, and young adults, they can still present at any age. The most frequent localization includes the trunk and extremities; however, they have also been described at the level of the mucous membranes [6,12,13]. PEMs are currently considered to be neoplasms with a low degree of malignancy by virtue of the rare ability to metastasize at a distance even in the presence of a high capacity to metastasize to the regional lymph nodes [6,13]. The conclusions are still far from being completely clear though; for example, in 2012, Christian Posch et al. reported their experience with regard to TMJ, considering the malignant potential of this entity suggested by mitotic index and necrosis to be so frequent that it was found that TMJ was considered a malignant variant of melanoma in all respects [9].

In 2015, Ritva Vyas et al. reported a systematic review with meta-analyzes relating to TMJ: 190 cases of TMJ were identified, which occurred with a similar frequency between men and women, and which mostly affected the Caucasian race (53.7%). The authors reported an average Breslow depth of 3.8 mm; ulceration was reported present in 15.8%; and dermal mitoses greater than or equal to 1/mm (2) were reported in 27.4%. Furthermore, 78 patients (55.7%) had undergone a wide local excision with sentinel lymph node biopsy, which was positive in 41.0% of cases. A total of 32 patients underwent completion lymph node dissection, with a 34.4% positivity rate. Locoregional recurrence was reported in 15 patients, recurrence with distant metastases in 6 patients, and death in 5 patients [14]. In 2017, Michael J Bax et al. reported their experience with nine TMJ/PEM patients at the University of Rochester over 10 years. Five patients underwent sentinel lymph node biopsy, with three of five having a positive sentinel lymph node. All nine patients were alive and disease-free with average follow-up of 38.75 months. Two tumors were tested for common melanoma-associated mutations, and were negative, except for a telomerase reverse transcriptase promoter deletion detected in 1 sample. The deletion has not been associated with melanoma, thus its biologic significance is still unclear [15,16,17]. Moreover, Urso et al., Avilés-Izquierdo, and Russo D. et al. reported their cases of ATM/PEM with clinical outcomes more or less similar to the previous cases mentioned [18,19,20]. In 2017, Ashley Tarasen et al. presented a very interesting report of two cases of PEM/TMJ occurring in a 63-year-old man and a 72-year-old woman and provided us with histological details of some importance. In detail, the first case related to a lesion that arose near the outer root sheath of the hair follicle, in the swelling region, where the stem cells reside. In the second case, however, the lesion had arisen from a previous intradermal nevus, suggesting the possibility that this lesion may have originated from this cell line [21]. On the other hand, M Kathryn Leonard et al. described in great detail how much the melanomas arising in animal species, such as mouse models, could be very aggressive and how the creation of genetically modified mouse models (GEMMs) can be of help to better understand the pathogenesis of primary melanoma and its potential therapy (CDK4 R24C, surviving, and NME1/NME2) [22].

In 2018, Robledo-Sánchez et al. [2] reported a rare case of ATM in a 79-year-old subject who died a few months later from metastatic dissemination. In particular, in recent years, an increasing amount of immunohistochemical and molecular evidence is trying to elucidate the mechanisms of etiopathogenesis; in detail, in two recent works [9,21,22,23,24], Cohen et al. have identified an absence of mutation in the codifying gene for the protein PRKAR1A in molecular terms, but demonstrated a loss of immunohistochemical expression of this marker, proposing a possible diagnostic aid for PEMs with respect to lesions that can mimic them as conventional, cellular, or malignant blue nevus and deep penetrating nevus (DPN). Additionally, a recent study showed the presence of GNAQ mutations in PEMs; thus, these studies provided molecular support for the classification of these tumors as variants or blue nevi [24].

## 5. Conclusions

In this work, we wanted to make our contribution in relation to an entity of exquisite anatomical-pathological interest, which still represents a diagnostic and therapeutic challenge today. One, albeit succinct, review of the literature has allowed us to analyze what have been the classification changes of this entity and how molecular biology can help in the correct definition.
